# Longitudinal bioimpedance assessments to evaluate hydration in POEMS syndrome

**DOI:** 10.1136/bmjspcare-2015-000991

**Published:** 2016-04-28

**Authors:** Amara Callistus Nwosu, Lauren Morris, Catriona Mayland, Stephen Mason, Andrew Pettitt, John Ellershaw

**Affiliations:** 1 Marie Curie Palliative Care Institute Liverpool (MCPCIL), University of Liverpool, Liverpool, UK; 2 Royal Liverpool and Broadgreen University Hospitals NHS Trust, Merseyside, UK

**Keywords:** Haematological disease, Quality of life, Clinical assessment, Clinical decisions

## Abstract

Polyneuropathy, organomegaly, endocrinopathy, M-protein and skin changes (POEMS) syndrome is a rare paraneoplastic disorder associated with an underlying plasma cell dyscrasia and multiorgan failure. POEMS syndrome is potentially fatal and adversely affects quality of life. Oedema is common with many patients affected by pleural effusions, ascites and lower limb oedema. Bioelectrical impedance vector analysis (BIVA) is a non-invasive assessment tool, which enables rapid bedside assessments of nutrition and hydration. This paper describes the use of sequential BIVA assessments to evaluate the response to diuretic therapy in a woman aged 52 years with POEMS syndrome. This case illustrates the potential to use BIVA to conduct longitudinal assessments of hydration status. This provides opportunities for further research using BIVA to monitor hydration and response to interventions. This may be useful in specific situations, for example at the end of life.

## Introduction

Polyneuropathy, organomegaly, endocrinopathy, M-protein and skin changes (POEMS) syndrome is a rare paraneoplastic disorder associated with an underlying plasma cell dyscrasia and multiorgan failure.^1^ The pathogenesis of POEMS syndrome is likely caused by overproduction of vascular endothelial growth factor (VEGF).^2^ POEMS syndrome is potentially fatal and adversely affects quality of life. Oedema is common with many patients affected by pleural effusions and ascites.^1^ ^2^

There is limited evidence to determine the association between hydration and symptoms in advanced cancer.^3^ Physical examination has a low sensitivity and specificity for identifying fluid deficit and, biochemical methods show little association with hydration status.^3^ ^4^ There is no routine hydration assessment method for patients with advanced cancer. The evidence for the efficacy of clinically assisted hydration (CAH) in advanced cancer is limited, conflicting and inconclusive. The subject of hydration is extremely important to patients and caregivers; there is concern about the risk of harm to patients through the use or non-use of CAH.^3^

Bioelectrical impedance vector analysis (BIVA) is a non-invasive, validated body composition assessment method, which may be useful in the assessment of hydration.^5^ To date, there are no published reports about the utility of BIVA in POEMS syndrome.

## Case history

This case describes the use of BIVA in a woman aged 52 years with POEMS syndrome. She had a history of two autologous stem cell transplants, renal impairment and recurrent lower limb and abdominal oedema. Oedema was a cause of great discomfort and had adversely affected her mobility. She was referred to the specialist palliative care team for symptom management and fluid assessment using BIVA. Following the baseline assessment, she received 40 mg of oral furosemide daily in combination with advice about fluid restriction. Two further BIVA assessments were conducted at weekly intervals following the commencement of diuretic therapy to assess the response to diuretic therapy. A clinical evaluation of peripheral oedema (upper and lower limbs) was also conducted during these assessments.

## Bioelectrical impedance vector analysis (BIVA)

Bioimpedance analysis involves a tetrapolar technique to deliver a single-frequency electrical current of 50 kHz. The technique works on the principle that fluid and cellular structures will provide different levels of resistance to an electrical current as it passes through the human body. Bioimpedance provides the following direct measurements: resistance (R) assessing cellular hydration, reactance (Xc) assessing tissue integrity and phase angle (PA), which is reported to be a useful indicator of health and prognosis.^5^ Bioimpedance analysis was conducted using the EFG-3 ElectroFluidGraph Vector Impedance Analyser (Akern) in line with methods and recommendations described elsewhere.^5^ Regression equations of the manufacturer (Akern BodyGram Pro 3.0) were used to calculate total body water (TBW), intracellular water (ICW) and extracellular water (ECW).^6^ These validated equations were derived from previous research.^7^

**Figure 1:**
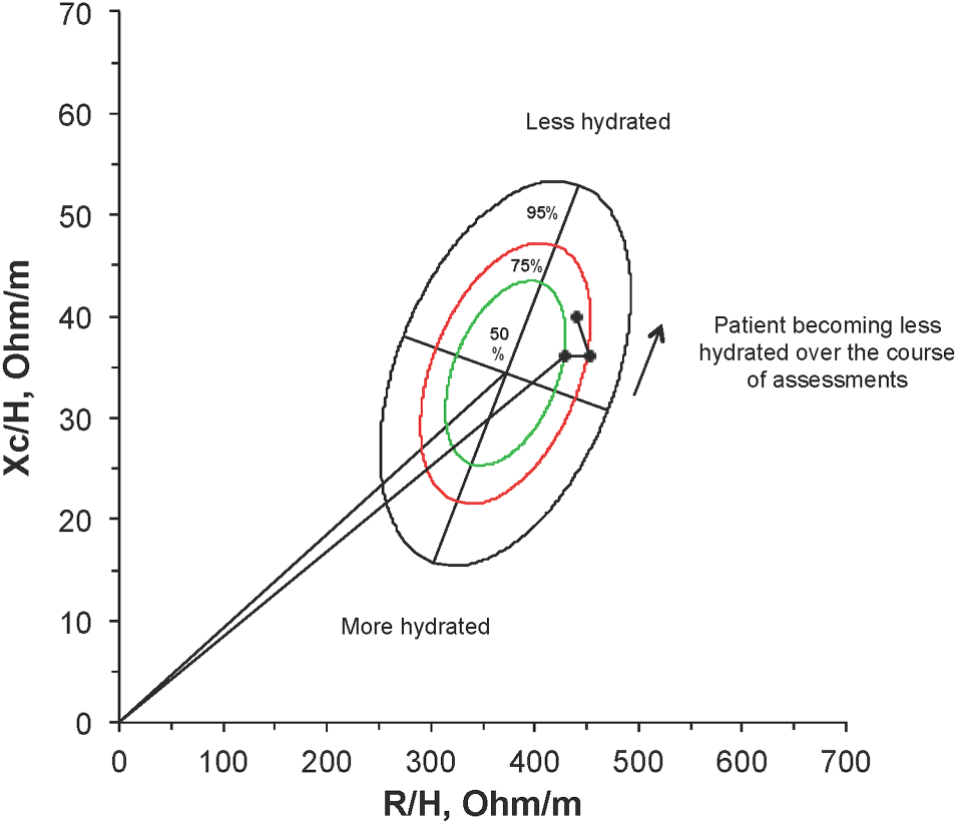
Longitudinal change of hydration represented by the BIVA RXc graph. The RXc graph method allows statistical analysis of bivariate distributions of successive impedance vectors of an individual relative to the 50%, 75% and 95% tolerance ellipses of a non-cancer reference population. The black dots represent the study assessments. The black arrow demonstrates the linear trend of the assessments. BIVA, bioelectrical impedance vector analysis; R, resistance; Xc, reactance.

BIVA enables interpretation of bioimpedance data, which is independent of regression equations and body weight. To establish BIVA, the direct impedance measurements (R and Xc) were plotted as a point (bivariate random vector) on a probability graph (RXc graph); this represented the sex-specific and race-specific tolerance intervals of a non-cancer reference population used for the analysis (figure 1).^8^

## Results

At baseline, BIVA demonstrated the participant's overall body composition was just outside the normal ellipse 50th centile and did not suggest fluid overload (figure 1). Following diuretic therapy, the subsequent assessments demonstrated a reduction in hydration volume. This corresponded with weight loss and a reduction in clinically detectable oedema. Throughout the assessments, TBW was low relative to body weight (table 1) and ECW was high relative to TBW.

**Table 1:** Results of bioelectrical impedance analysis

	Baseline	Follow-up 1	Follow-up 2	
Bioimpedance variables N (ohms)				
R (Ω)	679	715	696	
Xc (Ω)	57	57	63	
PA (Ω)	4.8	4.6	5.2	
Weight (kg)	69.5	67.7	66.4	
Height (cm)	157.9	157.9	157.9	
BMI (kg/m^2^)	27.9	27.1	27.0	
Hydration variables N (L)				Normal values in an adult woman
TBW	29.6 (43% of body weight)	28.6 (42% of body weight)	28.9 (44% of body weight)	45–50% of body weight
ICW	14.2 (48% of TBW)	13.3 (47% of TBW)	13.3 (47% of TBW)	55% of TBW
ECW	15.4 (52% of TBW)	15.3 (53% of TBW)	15.3 (53% of TBW)	45% of TBW
Clinical examination of oedema				
	Pitting oedema evident around ankles and shin	Small non-pitting oedema to ankle	No oedema detectable	

BMI, body mass index; ECW, extracellular water; ICW, intracellular water; PA, phase angle; R, resistance; TBW, total body water; Xc, reactance.

## Discussion

### Main findings

BIVA demonstrated a reduction in hydration volume following the intervention (diuretic therapy and fluid restriction). This corresponded clinically with weight loss and a reduction in clinically detectable oedema.

### Strengths

To our knowledge, this is the first study to use BIVA to evaluate hydration in POEMS syndrome. Furthermore, the evaluation of longitudinal change in hydration in POEMS syndrome using BIVA, following intervention with diuretics, is novel. The advantage of BIVA is that it allows information to be obtained simultaneously about changes in tissue hydration or soft tissue mass, independent of regression equations, or body weight. This allows for accurate interpretation of BIVA readings even if patients are at extremes of weight or volume distribution.

### Significance of the findings and comparison with previous studies

The correlations between hydration change, body weight and observable oedema are consistent with non-cancer studies using BIVA to measure hydration.^9^ The participant commented on the simplicity of the BIVA method. Her experience is consistent with the findings of our previous research using BIVA to evaluate hydration in advanced cancer, which adds to the evidence that the BIVA method is not burdensome in palliative care.^10^

TBW was low relative to body weight; however, ECW was high compared with TBW. This suggests an imbalance of ECW:ICW ratio, which may be the potential cause of the oedema. Although there was a clinical reduction in weight and oedema, this was not reflected by change in the ECW volume. The reason for this is not clear; however, a potential explanation may be that the bioimpedance regression equations lack the sensitivity to detect small changes in fluid volume.

### Limitations

Bioimpedance regression equations are limited in cancer owing to their reliance on physiological assumptions (which include requirements about body shape, the relationship between trunk and leg lengths, soft tissue hydration level and fat fraction).^5^ Although the equations have been validated against reference methods with good accuracy, they may lack accuracy in situations where basic assumptions of the bioimpedance method are not met (eg, in cancer or at extremes of body mass and hydration).^5^ The limitations of regression equations are overcome by BIVA, which is independent of the regression analysis and body weight. We acknowledge that we are unable to account for potential confounding factors, which may have influenced hydration between assessments. Furthermore, the relative under-representation of the trunk by whole-body impedance limits our ability to quantify or localise the oedema.^5^ Consequently, BIVA should be interpreted with acknowledgement of the clinical presentation of the individual.

### Implications for practice

The properties of BIVA (non-invasive, safe, accurate and painless) highlight its potential to assess hydration in palliative care. BIVA could potentially be used to measure hydration in specific clinical scenarios (eg, bowel obstruction, vomiting and diarrhoea) or to monitor the response to interventions (eg, CAH).^9^ In cancer, BIVA has advantages over regression equations; however, the results should be interpreted carefully with knowledge of the clinical presentation of the patient.

### Research opportunities

In research, BIVA can be used to monitor hydration in studies that evaluate the efficacy of CAH. Furthermore, longitudinal hydration assessments could be conducted at specific phases of illness (eg, to examine the relationship between symptomatic burden and clinical hydration states in the last hours and days of life).

## Conclusion

This case report highlights the potential to use BIVA to monitor hydration states over time in response to interventions. BIVA overcomes the limitations of bioimpedance regression equations. More research is needed to determine the potential of BIVA to improve the evaluation and management of hydration states in advanced cancer.
